# Electron yield soft X-ray photoabsorption spectroscopy under normal ambient-pressure conditions

**DOI:** 10.1107/S0909049513003592

**Published:** 2013-03-16

**Authors:** Yusuke Tamenori

**Affiliations:** a Japan Synchrotron Radiation Research Institute/SPring-8, 1-1-1 Kouto, Sayo, Hyogo 679-5198, Japan

**Keywords:** soft X-ray photoabsorption spectroscopy, normal ambient pressure, electron yield detection, conversion electron yield detection, hydration

## Abstract

Ambient-pressure soft X-ray photoabsorption spectroscopy (XAS) was demonstrated to be applicable to the chemical analysis of hydrated transition-metal compounds. For this purpose, even under ambient-pressure conditions, electron yield detection XAS (EY-XAS), based on a simple drain-current set-up, was used to overcome a weakness in fluorescence yield detection XAS (FY-XAS), which does not give a pure soft XAS. The feasibility of EY-XAS was investigated and it was clarified that the EY-XAS under ambient-pressure conditions corresponds to the mixed data of the total EY and conversion EY spectra. Normal ambient-pressure EY-XAS analysis was applied to anhydrous (CoCl_2_) and to hydrated (CoCl_2_·6H_2_O) cobalt chloride at the Co *L*_23_-edge. The present measurements demonstrated the ability to unambiguously distinguish the different chemical states of cobalt ions, relying upon spectral differences that indicate octahedral/quasi-octahedral structural changes as a result of hydration/dehydration reactions.

## Abstract 2

Soft X-ray photoabsorption spectroscopy under normal ambient-pressure conditions using electron yield detection was demonstrated. This technique provided unambiguous photoabsorption data for hydrated transition-metal compounds and identified the different chemical states of cobalt ions.

## 1. Introduction   

X-ray photoabsorption spectroscopy (XAS), which is often called X-ray absorption fine-structure (XAFS) spectroscopy, is one of the best techniques for the determination of the electronic structures of materials (Stöhr, 1992 ▶). One of the advantage of XAS analysis is that samples can be analyzed regardless of their physical state (solid, gas or liquid), and it is not restricted to the analysis of certain elements, as are Mössbauer and nuclear magnetic resonance (NMR) analysis. Furthermore, because the core electrons excited by X-ray absorption are localized to the atoms from which they originate, X-ray core-electron transitions allow investigation of the chemical properties of specific elements in complex materials. In particular, XAS analyses using soft X-rays are uniquely appropriate not only for studying light elements but also for studying transition metals (Zaanen *et al.*, 1985 ▶; de Groot & Kotani, 2008 ▶). Since XAS is governed by dipole selection rules, the *d*-shell properties of first-row transition metals are best probed by the *L*_23_-edge absorption (2*p*–3*d* transition) in the soft X-ray region.

However, so far the application of soft X-ray analysis has been highly restricted. Since soft X-rays below 2.0 keV are largely absorbed by air under ambient pressure, it is standard procedure to perform soft X-ray analysis under high vacuum. Although the high-vacuum chamber produces an ideally clean environment for the sample, chemically significant phenomena take place under ambient pressure, such as the coexistence of water and oxygen gas. One of the active areas of modern research in soft X-ray analysis is to improve the versatility of the sample environment. For example, vacuum-compatible sample holders containing a liquid cell have been developed for the analysis of liquid materials (Guo *et al.*, 2004 ▶; Liu *et al.*, 2007 ▶; Schreck *et al.*, 2011 ▶) and for the observation of chemical reactions (de Smit *et al.*, 2008 ▶; de Groot *et al.*, 2010 ▶; Huse *et al.*, 2010 ▶; Kolmakov *et al.*, 2012 ▶).

Ideally, it would be convenient for researchers to be able to apply soft X-ray analytical techniques to their samples without using high-vacuum chambers and special vacuum-compatible reaction cells. One solution is to use a helium path because helium gas has a higher transmission than air in the soft X-ray region as a result of its smaller atomic number. Since the pioneering work by Roper *et al.* in 1992, several investigations have been reported to obtain reliable spectra under atmospheric pressure conditions using soft X-rays as a probe (Roper *et al.*, 1992 ▶; Yagi *et al.*, 2004 ▶; Nakanishi *et al.*, 2010 ▶). In this scenario, the high vacuum of the light source or synchrotron radiation beam is protected by isolating the analysis chamber using a vacuum window. For example, Nakanishi *et al.* (2010 ▶) have reported their recent successful XAS analysis of hydrated compounds at the Mg *K*-edge region (1.3 keV) using a thin Be foil (15 µm) as a vacuum window. However, soft X-ray analysis under atmospheric pressure is still limited to energies higher than 1 keV, since photoabsorption by the window reduces the photon flux arriving at the sample. To overcome the current limitations, we developed a new approach to XAS analysis using a differential pump instead of a vacuum window (Tamenori, 2010 ▶). We specifically focused on the application of lower-energy soft X-rays (below 1 keV) under normal ambient-pressure conditions, the range in which the *K*-edges of carbon and oxygen and the *L*_23_-edges of 3*d* transition metals are found.

Another issue to be solved is the development of an XAS signal detection technique instead of fluorescence detection. Fluorescence yield (FY) detection, which is the more common XAS signal detection method under high pressure, has two drawbacks. One is the spectral line shape distortion, which is well known as the ‘self-absorption effect’ (Jaklevic *et al.*, 1977 ▶; Zschech *et al.*, 1992 ▶; Meitzner & Fischer, 2002 ▶). Another issue is that FY measurements do not produce pure XAS, when multiplet effects are important, because the fluorescence decay probability strongly depends on the final state (de Groot *et al.*, 1994 ▶). In contrast to fluorescence decay, the Auger decay probability is approximately constant. Therefore, even under normal ambient-pressure conditions, the application of electron yield (EY) measurements is indispensable to obtaining reliable XAS in the soft X-ray region.

In the present research, we demonstrate the capabilities of normal ambient-pressure soft XAS using EY detection. This technique was used for XAS measurements of the coordination states in hydrated transition-metal compounds. We observed a change in the coordination states of cobalt ions in cobalt(II) chloride *via* hydration/dehydration using *L*_23_-edge XAS analysis. Such hydrated compounds adopt different crystal structures from those of their dehydrated counterparts and, consequently, generate distinct XAS. This technique provides information about the symmetry and oxidation state of the analyte and allows for the identification of unknown species, establishing it as a powerful tool for speciation analysis of transition metals under normal ambient-pressure conditions.

## 2. Experimental   

### 2.1. Soft X-ray source   

The normal ambient-pressure soft XAS set-up was installed at the c-branch of the soft X-ray photochemistry beamline (BL27SU) of SPring-8. Radiation from a figure-8 undulator was dispersed by a soft X-ray monochromator with varied-line-spacing plane gratings, and then introduced into the XAS instrument (Tanaka *et al.*, 1998 ▶; Ohashi *et al.*, 2001 ▶). XAS were obtained by scanning the undulator gap as well as the monochromator to maintain maximum intensity of the incident soft X-rays, and by scanning the width of the entrance and exit slits to maintain constant resolving power. The photon-energy-resolving power was set to *E*/Δ*E* = 5000. The X-ray beam diameter was focused to a 200 µm spot on the sample. During analysis, the intensity of the incoming photon beam was monitored by measuring the drain current on the surface of a post-focusing mirror.

### 2.2. Ambient-pressure soft X-ray spectroscopy set-up   

**Figure 1:**
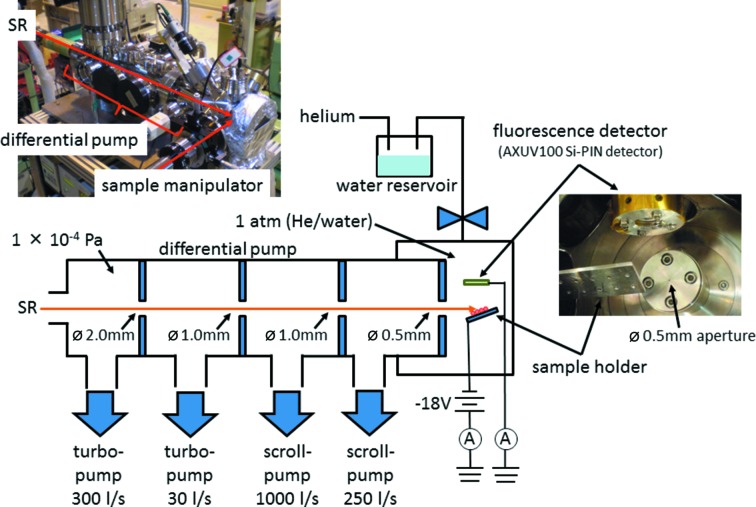
Schematic diagram of the normal ambient-pressure XAS apparatus. Inset photographs show pictures of the whole system (top left) and the inside of the analysis region taken from the downstream of the system (right).

A schematic diagram of the experimental set-up is shown in Fig. 1 ▶. The XAS chamber was connected to the beamline *via* a four-stage differential pump (Tamenori, 2010 ▶). When the sample chamber was filled with helium (1 atm), the pressure at the uppermost stage of the differential pump was maintained at 1.0 × 10^−4^ Pa. The system provided enough isolation between the sample chamber atmospheric pressure and the high vacuum of the beamline across a short distance, eliminating the need for a vacuum window for the soft X-ray beamline. The loss of photon flux in the differential pump section was less than 5% at 800 eV, roughly one-sixth of the loss observed when using a 200 nm Si_3_N_4_ vacuum window. The total transmission at 800 eV (at the cobalt *L*_23_-edge) measured 50 mm downstream from the last aperture (diameter 0.5 mm) was about 90%. Additionally, there was no steep reduction in photon intensity at the absorption edge of the elements contained in the vacuum window, providing ideal conditions for XAS analysis. The pressure of the analysis region could be reduced to 1.0 × 10^0^ Pa *via* evacuation through the aperture of the differential pump, and pure helium gas was installed to produce the normal ambient-pressure conditions.

Evaporation of water from the sample was significantly reduced under normal ambient pressure compared with in a vacuum. However, even under conditions of 1 atm of helium, drying of the sample could not be prevented completely. Therefore, in order to keep the sample hydrated, a helium–water mixed gas was introduced into the XAS sample chamber. During analysis of the hydrated samples, helium gas was passed through a reservoir containing purified water at room temperature, and the mixed gas was introduced through a nozzle with an inner diameter of 6.25 mm. The moisture level of the sample chamber was monitored by the color of the CoCl_2_ sample. Because the hydration/dehydration reaction is fast and is accompanied by a change in color, cobalt chloride is used as a moisture indicator in drying agents (Solomon, 1945 ▶). CoCl_2_·6H_2_O is deep purple in color, whereas the anhydrous form is sky blue (Grime & Santos, 1934 ▶).

### 2.3. XAS measurements   

XAS were obtained using both EY and FY detection. EY-XAS were recorded using the same experimental set-up as that for conventional sample drain-current measurements. The aluminium sample holder was biased (−18 V) by two dry batteries (9 V) with respect to the analysis chamber, and the drain current was monitored by an ammeter. The FY-XAS were recorded using a Si photodiode detector (AXUV-100; IRD), which was installed in front of the sample (Fig. 1 ▶). EY-XAS and FY-XAS were recorded simultaneously for a sequence of incident X-ray energies in the usual fashion used for XAS measurements. Monochromatic light was irradiated at an angle of ∼45° relative to the sample normal. Data analysis to remove the background and qualitatively analyze the XAS was carried out manually. The data were normalized for variations in the primary X-ray intensity. A linear pre-edge was removed for each spectrum and the data were normalized by the height of the edge jump.

### 2.4. Sample preparation   

In the present research, we chose a colored silica gel ball as a sample, since it is easier to check the color of the sample and to control hydrating/dehydrating conditions than it is using pure CoCl_2_ powder. Anhydrous colored silica gel (CoCl_2_/SiO_2_) was purchased from Hakuyo. The concentration of CoCl_2_ in the silica gel was 4%. The hydrated sample was prepared by immersing the anhydrous sample in purified water for 1 h. Purified water was obtained using a water purification system (Barinsted EASYpure RF).

As a standard material, high-purity cobalt(II) chloride hexahydrate (CoCl_2_·6H_2_O, 99.3% purity) was purchased from MP Biomedicals, and anhydrous cobalt(II) chloride (CoCl_2_, 99.4% purity) was purchased from Wako Pure Chemical Industries. The standard samples of CoO and LiCoO_2_ were purchased from Wako Pure Chemical Industries and Kishida Chemical Company, respectively. All of the above materials were used without further purification. Samples were fixed with conductive double-sided carbon tape onto an aluminium sample holder and installed in the XAS chamber.

### 2.5. Atomic multiplet calculations   

Atomic multiple simulations for Co(II) were performed using the *CTM4XAS 5.0* program, including full spin–orbit coupling and crystal-field effects (Cowan, 1981 ▶; Stavitski & Groot, 2010 ▶). The simulation default values were determined by referring to the literature concerning cobalt ions of the same oxidation state and symmetry, and optimized by repeated simulations (de Groot *et al.*, 1993 ▶; Papaefthimiou *et al.*, 2011 ▶).

## 3. Results and discussion   

### 3.1. EY-XAS measurements under normal ambient-pressure conditions   

**Figure 2:**
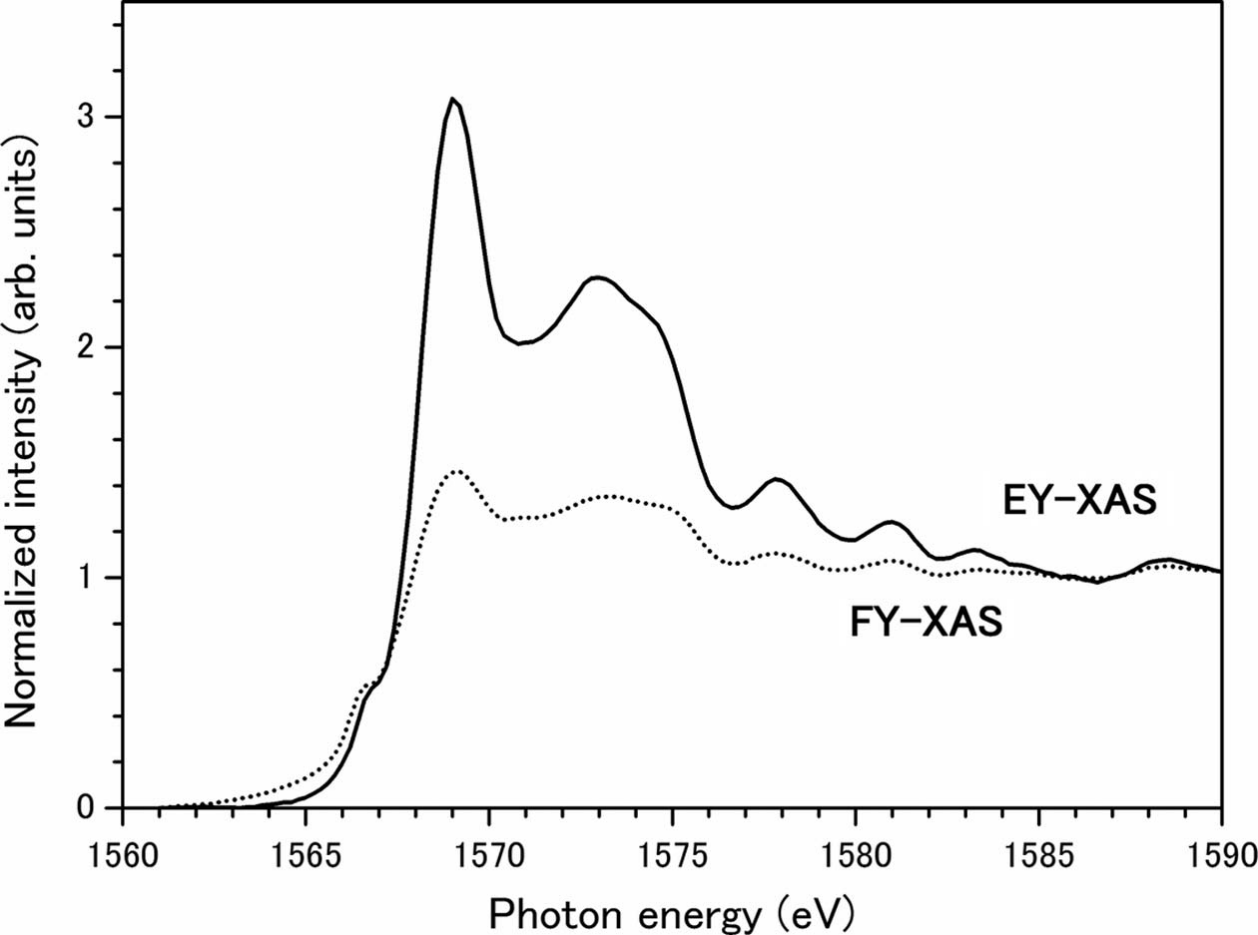
EY- (solid line) and FY-XAS (dashed line) of 2 mm-thick α-Al_2_O_3_ obtained in the Al *K*-edge region. Both spectra were obtained under normal ambient-pressure conditions (1 atm helium path), and were obtained by simultaneous measurements. The spectra were subjected to background subtraction and then normalized by the edge-jump height.

The feasibility of EY detection under normal ambient-pressure conditions was confirmed. Fig. 2 ▶ shows the XAS of 2 mm-thick α-Al_2_O_3_ obtained using the EY-XAS (solid line) and FY-XAS (dashed line) techniques in the Al *K*-edge region. The indicated spectra were obtained by background subtraction and then normalized by the edge-jump height. One significant feature of these measurements is that the XAS can be obtained by the EY method, even though α-Al_2_O_3_ is an insulating material. FY detection is insensitive to charging of the sample, making it applicable for measuring the XAS of insulating materials, but XAS measurements using a conventional drain-current method under vacuum conditions are inhibited by sample charging.

Here, the question of how the EY is detected by this experimental set-up arises. The EY-XAS in Fig. 2 ▶ was obtained with the same set-up as that used in the conventional sample drain-current method. Since a thick full insulator is used as the sample, direct detection of the sample drain-current can be ruled out. Another possibility is that the positive ions are collected by the sample holder. Because the probabilities of ion desorption by soft X-ray absorption are negligible, the production of secondary ions is considered to be an important process. This is similar to well known processes such as conversion EY (CEY) detection in the hard X-ray region (Kordesch & Hoffman, 1984 ▶; Tourillon *et al.*, 1987 ▶). A conceivable EY detection scheme is as follows. Auger electrons and X-ray fluorescence, which are eliminated in the core-hole decay processes, ionize the surrounding helium gas, and secondary ions can be collected by the negatively biased sample holder. The collected ions are neutralized on the surface of the sample holder, and the number of lost electrons is measured as the drain current. In order to confirm the proposed model, the effects on the sample current of positive and negative bias voltages were investigated by changing the polarities of the dry batteries. Reversing the bias voltage reversed the polarity of the signal. This result can be interpreted as the positively biased sample holder collecting eliminated and secondary electrons.

The EY-XAS measurements of insulating samples without sample charging can also be interpreted by considering the CEY detection scheme. Soft X-ray irradiation eliminates electrons from the sample surface, and positive charges will be distributed on the sample surface. Under vacuum conditions, distortion of the spectral shape should occur as a result of the accumulation of positive charges on the sample surface. On the other hand, under low-vacuum or ambient-pressure conditions, the photoelectrons and Auger electrons emitted from the sample ionize the surrounding helium gas, and this produces several secondary electrons. These electrons are flooded around the sample surface and neutralize the positive surface charges. Similar phenomena are well known in secondary electron microscopy (SEM) measurements as low-vacuum SEM (Moncrieff *et al.*, 1978 ▶).

The XAS obtained using two different detection modes show different spectral profiles. The peak height at the white line (1569.0 eV) is 1.45 in the FY-XAS, whereas in the EY-XAS it is 3.08. The clear peak intensity reduction in the FY-XAS is known to be caused by the ‘self-absorption effect’ (Jaklevic *et al.*, 1977 ▶; Zschech *et al.*, 1992 ▶; Meitzner & Fischer, 2002 ▶). Under ambient-pressure conditions, EY detection produces more reliable XAS than does FY detection. On the other hand, Nakanishi & Ohta (2008 ▶) determined that the peak height at the white line is 5.4 by total EY (TEY) measurements using a microchannel-plate detector. The obtained peak height of the EY-XAS (3.08) is lower than those reported in earlier papers. This result suggests that the EY-XAS measurement was also affected by the saturation effect.

Zheng *et al.* (1997 ▶) indicated that the probing depth of the CEY method is deeper than that of the TEY method. This phenomenon, caused by the escape depth of electrons in solid materials, strongly depends on the kinetic energies of these electrons. The TEY signal is dominated by low-energy electrons with kinetic energies below about 20 eV, and the mean free paths of these electrons are shorter than 5 nm (Stöhr, 1992 ▶). However, these low-energy electrons cannot contribute to the production of helium ions, since the kinetic energies of most secondary electrons are not enough to ionize helium atoms [24.58 eV (Siegbahn *et al.*, 1969 ▶)]. In CEY detection, higher-energy electrons such as Auger electrons can contribute to the production of secondary ions, and these electrons are eliminated from much deeper inside the sample. As a result, the XAS obtained by the CEY technique is more sensitive to the self-absorption effect than is that obtained by the TEY technique.

### 3.2. EY-XAS measurement of dehydrated CoCl_2_ under various pressure conditions   

From the EY-XAS measurement of an insulating material, the contribution of the CEY signal to the EY-XAS measurement was confirmed. On the other hand, a conductive sample, which can measure the sample drain-current, should produce identical data to those obtained by TEY detection, even under ambient-pressure conditions. This assumption will be experimentally confirmed in this section.

**Figure 3:**
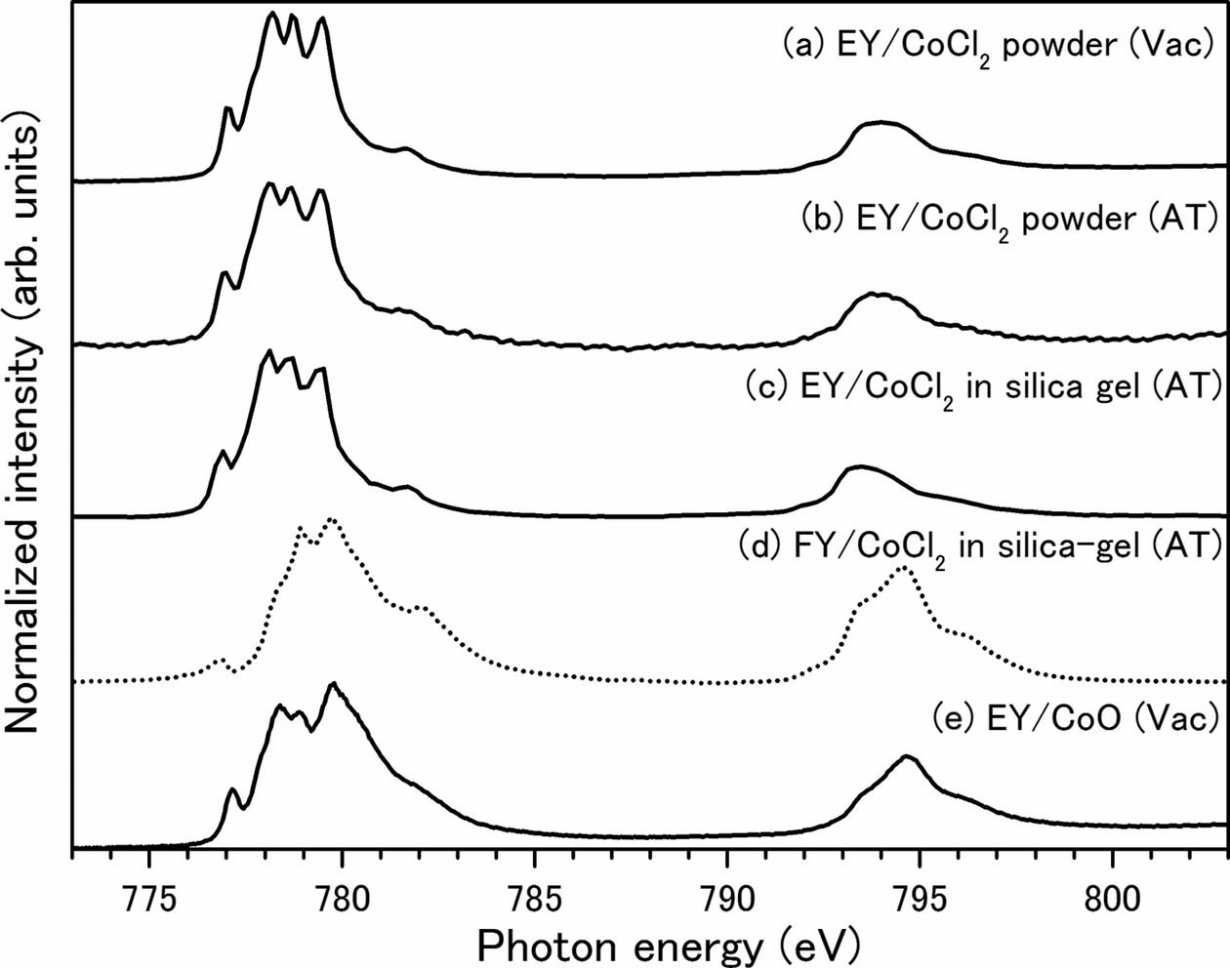
Co *L*_23_-edge photoabsorption spectra of Co compounds under various pressure conditions: (*a*) EY-XAS of dehydrated CoCl_2_ powder, measured under high vacuum (1 × 10^−5^ Pa), (*b*) EY-XAS of dehydrated CoCl_2_ powder, measured under normal ambient-pressure conditions, (*c*) EY-XAS of dehydrated CoCl_2_ in silica gel, measured under normal ambient-pressure conditions, (*d*) FY-XAS of dehydrated CoCl_2_ in silica gel, measured under normal ambient-pressure conditions; the spectra in (*c*) and (*d*) were simultaneously observed. (*e*) EY-XAS of dehydrated CoO powder, measured under high vacuum as a standard material. XAS obtained by EY detection are indicated by solid lines and that by FY detection is indicated by a dashed line.

Figs. 3(*a*)–3(*d*) ▶ show XAS of dehydrated CoCl_2_ samples obtained at the Co *L*_23_-edge under various pressure conditions. To understand the spectral profiles, the XAS of a standard material (CoO) is also shown in Fig. 3(*e*) ▶. Figs. 3(*a*) and 3(*b*) ▶ show the EY-XAS for pure CoCl_2_ powder under a high vacuum (1 × 10^−5^ Pa) and normal ambient atmospheric pressure conditions, respectively. The *L*_23_-edge XAS contain two regions (*L*_2_ and *L*_3_) arising from spin–orbit splitting of the 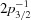 and 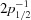 core holes. These spectra are almost identical, and the identical XAS obtained under different pressure conditions suggests two facts. The first is that the high pressure caused by the surrounding helium gas does not affect the chemical and electronic properties of the sample molecules. The second is that TEY detection is achievable for conductive samples, even under ambient-pressure conditions, as described above. As a result, the present measurements clarified that the EY-XAS under ambient-pressure conditions corresponds to the mixed data of the TEY and CEY spectra. For conductive materials, the EY-XAS measurements mainly correspond to the data obtained by TEY detection, whereas the contribution of CEY detection becomes significant for insulating materials, and is affected by the saturation effect. However, even for XAS measurements of insulating materials, the obtained EY-XAS was more reliable than the FY-XAS measurements. Therefore, EY-XAS measurements are applicable in XAS measurements under ambient-pressure conditions.

Fig. 3(*c*) ▶ shows the EY-XAS for a colored desiccant under atmospheric pressure conditions. This spectrum is identical to the top two spectra, and suggests that CoCl_2_ contained in the silica gel ball is identical to the pure CoCl_2_ reagent. This result indicates that, although silica gel is an insulating material, the saturation effect is negligible in the present EY-XAS because of the low concentration of silica gel. Fig. 3(*d*) ▶ shows the FY-XAS of a colored desiccant under atmospheric pressure conditions, as a typical spectrum obtained by FY detection. The FY-XAS obtained under the top two experimental conditions were also almost identical to the FY-XAS of the colored desiccant (data not shown). The spectral profile obtained by FY detection is clearly different from that obtained by EY detection. The intensity of the *L*_3_ band (in the 776–778 eV range) decreased in the FY-XAS. The area intensity ratio of the *L*_2_:*L*_3_ band in the EY-XAS is 1:3.1, whereas that of FY-XAS is 1:1.8. A similar phenomenon was indicated in earlier reports, and is interpreted as suppression of the *L*_3_ band intensity by the saturation effect (Liu *et al.*, 2007 ▶). Furthermore, depletion of some specific peaks can be confirmed in the multiplet structures. For example, the first and second peaks at 776.9 and 778.1 eV are clearly suppressed in the FY-XAS. This phenomenon seems to arise because the fluorescence decay probabilities strongly depend on the final states (de Groot *et al.*, 1994 ▶). The elimination of fluorescence X-rays is expected to be suppressed on excitation of these final states. The present results also demonstrate that EY-XAS measurements are applicable even under normal ambient-pressure conditions, and produce more reliable XAS data than do FY-XAS measurements.

Fig. 3(*e*) ▶ shows the EY-XAS of CoO as a reference material corresponding to dehydrous CoCl_2_. The XAS of anhydrous CoCl_2_ was found to be similar to that of CoO, indicating that the oxidation states of the cobalt ions as well as the symmetries of the CoCl_2_ crystals are analogous to those of CoO. In CoO, the Co(II) ions are coordinated to oxygen anions with octahedral geometry and *O*_*h*_ symmetry (de Groot *et al.*, 1993 ▶). This interpretation is consistent with previous X-ray diffraction investigations of CoCl_2_ crystals (Grime & Santos, 1934 ▶). The chlorine atoms adopt a cubic close-packed arrangement with the cobalt atoms located at the centers of octahedral groups of chlorine atoms.

### 3.3. Comparison of EY-XAS of unhydrated and hydrated CoCl_2_   

**Figure 4:**
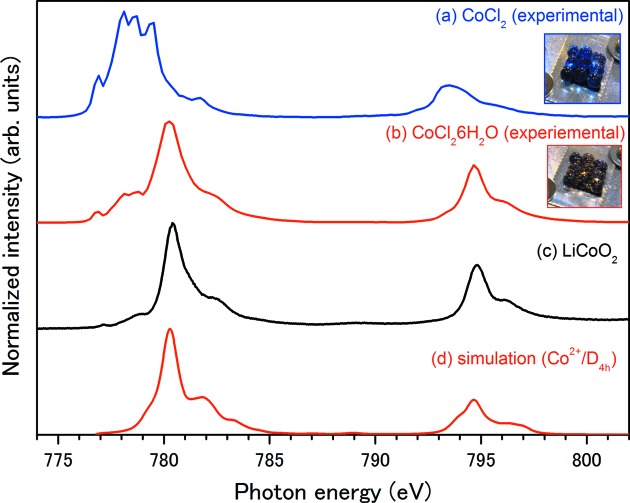
Co *L*_23_-edge photoabsorption spectra of Co compounds under various pressure conditions: (*a*) EY-XAS of dehydrated CoCl_2_ in silica gel, measured under 1 atm helium condition, (*b*) EY-XAS of hydrated silica gel (*i.e.* CoCl_2_·6H_2_O), measured under 1 atm helium/water mixture conditions, (*c*) EY-XAS of LiCoO_2_ powder, measured under high vacuum, as a standard material, and (*d*) spectrum from ligand-field multiplet simulation of cobalt(II) ions with *D*_4*h*_ symmetry.

Figs. 4(*a*) and 4(*b*) ▶ show the EY-XAS for a colored desiccant under normal ambient-pressure conditions. The former is the spectrum obtained for dehydrated CoCl_2_, which is sky blue, recorded under 1 atm of pure helium. The latter is the spectrum of CoCl_2_ hydrate, which is deep purple, recorded under 1 atm of a mixed helium/water gas. In order to confirm the sample conditions, photographs of the samples are shown as insets. Of particular interest are the spectral profiles of the hydrated and dehydrated CoCl_2_ obtained using EY-XAS, which differ substantially. The intensities of the multiplet peaks in the range 777–779 eV decreased in the XAS of CoCl_2_·6H_2_O, whereas the peak at 780.5 eV was enhanced. Since both measurements were performed under high-pressure conditions, the possibility that the high pressure changes the electronic structure of the sample can be ruled out. Another interpretation of the spectral change is a change in the valence or molecular structure as a result of hydration. The pronounced differences in the line shapes suggest that the local structures and/or the oxidation states of the cobalt ions are significantly different in the two samples.

The XAS of CoCl_2_·6H_2_O and standard trivalent cobalt compounds such as LiCoO_2_ are very similar (Fig. 4*c* ▶). At first glance, this agreement suggests that the oxidation state changed from divalent to trivalent. However, this is unlikely because of the instability of trivalent cobalt ions. Although cobalt(III) is stable in the presence of a complexing agent such as ammonia, in an aqueous solution containing no complexing agent cobalt(III) is easily reduced to cobalt(II) (Rajbir, 2002 ▶). The impurities contained in the silica gel could act as complexing agents and could affect the oxidation state of the cobalt during the hydration/dehydration processes. However, this scenario can be ruled out because the change in the spectral profile was replicated by XAS analysis of pure CoCl_2_ (data not shown). Therefore, we conclude that it is unlikely that water molecules added by hydration oxidized the cobalt ions in CoCl_2_·6H_2_O.

Another interpretation is that the crystal symmetry is changed by hydration. X-ray diffraction studies suggested the following crystal structure for CoCl_2_·6H_2_O (Mizuno *et al.*, 1959 ▶; Mizuno, 1960 ▶; El Saffar, 1960 ▶; Waizumi *et al.*, 1990 ▶; Souissi & Kammoun, 2011 ▶): two Cl^−^ ions and four water molecules are coordinated to a Co^2+^ ion in an octahedral geometry to form a CoCl_2_·4H_2_O group, whereas the other two water molecules of the formula unit are relatively free, and the groups are joined to one another by hydrogen bonds (O⋯H—O—H⋯O and O—H⋯Cl). The CoCl_2_·6H_2_O crystal has a quasi-octahedral geometry, and the complex unit belongs to the point-group *D*_4*h*_ symmetry. Based on these X-ray diffraction reports, we can assume that the symmetry of the CoCl_2_ crystal changed from *O*_*h*_ to *D*_4*h*_ as a result of hydration.

This hypothesis has been confirmed by ligand-field multiplet simulation (de Groot *et al.*, 1993 ▶; Papaefthimiou *et al.*, 2011 ▶). Fig. 4(*d*) ▶ shows the simulated spectra, assuming a *D*_4*h*_ symmetry with a ligand-field strength of 10*Dp*/*dt*/*ds* = 1.2/−0.5/0.1 eV for the divalent cobalt ion. The simulations faithfully reproduce the main spectral line profiles, whereas the spectrum from the simulation does not reproduce the lower-energy sides of the *L*_2_ and *L*_3_ bands. The discrepancy can be interpreted as contamination of the dehydrated CoCl_2_ signal. For example, the small peaks observed in the range 777–779 eV could not be reproduced in the simulated spectrum. However, these peaks correspond to the first three peaks observed in the spectrum of dehydrated CoCl_2_ (Fig. 4*a* ▶). Therefore, the discrepancy can be interpreted as partly dried CoCl_2_ being contained in the colored silica gel.

Furthermore, our explanation is also strengthened by the repeatable nature of the XAS analysis. After measurement of a hydrated sample, we stopped the helium/water mixed gas flow. This procedure evacuated the sample region and the CoCl_2_ underwent dehydration. Dehydration of the sample can be unambiguously confirmed by the change in the sample color (a color change from purple to blue). When we performed XAS measurements on this dehydrated sample, the spectrum indicated that the sample possessed *O*_*h*_ symmetry. Once the sample was rehydrated by immersion in water (undergoing a color change from blue to purple), subsequent XAS analysis revealed the *D*_4*h*_ nature of the analyte. The repeatable change in the XAS is achieved through the hydration and dehydration reactions alone, without adding a special oxidizing or reducing agent. Therefore, although the XAS of CoCl_2_·6H_2_O mimics the XAS of standard trivalent materials, we can eliminate the possibility that the spectral changes originate from a change in the oxidation state of the cobalt ion. We can conclude that the valence of the cobalt ion in CoCl_2_·6H_2_O is divalent and the crystal has *D*_4*h*_ symmetry.

## 4. Conclusions   

In the present study, it was demonstrated that EY soft XAS under normal ambient-pressure conditions can be applied by using the same layout as that with a normal sample drain-current used in conventional soft X-ray analysis. The feasibility of this method was confirmed by XAS measurements of Al_2_O_3_. EY XAS can be understood to have the mixed data of the total EY and conversion EY spectra. The drawback of EY-XAS under ambient conditions is that the obtained spectrum contains CEY signals. The CEY signals are enhanced on the measurement of insulating materials, and this causes spectral suppression as a result of the self-absorption effect. Despite this drawback, the obtained spectrum was more reliable than that using FY-XAS measurements, and is applicable in XAS measurements under ambient-pressure conditions.

EY-XAS analysis was applied to the analysis of cobalt ions in anhydrous CoCl_2_ crystals and to hydrated CoCl_2_ crystals under normal ambient-pressure conditions. The EY-XAS results unambiguously distinguished the chemical states of the cobalt ions in each sample. XAS simulation was used to show that the CoCl_2_ crystal underwent an octahedral/quasi-octahedral structural change by a hydration/dehydration process. We have successfully demonstrated that EY-XAS analysis under ambient pressure, using a helium path, is applicable to chemical state analysis, even for hydrated samples. This represents a significant milestone in the development of XAS analysis using soft X-rays under ambient conditions.
